# First Detection of Usutu Virus in Harbor Seals (*Phoca vitulina*)

**DOI:** 10.3390/v18030371

**Published:** 2026-03-17

**Authors:** Anne Schwarzer, Franziska Schopf, Insa Dammann, Katharina Kramer, Tanja Rosenberger, Christine Fast, Michaela Geese, Martin H. Groschup, Balal Sadeghi, Ute Ziegler

**Affiliations:** 1 Institute of Novel and Emerging Infectious Diseases, Friedrich-Loeffler-Institut, Federal Research Institute for Animal Health, Südufer 10, 17493 Greifswald-Insel Riems, Germany; 2 Landeslabor Schleswig-Holstein, Max-Eyth-Straße 5, 24537 Neumünster, Germany; 3 Seehundstation Friedrichskoog, An der Seeschleuse 4, 25718 Friedrichskoog, Germany

**Keywords:** Usutu virus, harbor seal, case report, Germany, Flavivirus, marine mammal, clinical manifestation, whole-genome sequencing

## Abstract

The zoonotic *Orthoflavivirus* Usutu virus (USUV) is distributed throughout Germany, putting hosts at a considerable risk of infection nationwide. Besides birds as reservoir hosts, a broad range of accidental hosts is suspected. However, there are few reports documenting the progression of USUV-associated disease. This case report describes the course of fatal USUV infections in three harbor seals (*Phoca vitulina*) from a rescue center on the North Sea coast in Germany. Corresponding samples were analyzed using (histo-)pathological, immunohistochemical, molecular and phylogenetic methods. The most prevalent findings in clinically affected animals were neurological signs and non-suppurative encephalitis. All animals were found dead or had been euthanized due to animal welfare reasons within 30 h after the onset of clinical signs. Blood samples taken from another 37 young harbor seals from the same rescue center in the same year revealed two further asymptomatic USUV RNA and antibody-positive animals. The sequences were found to belong to USUV lineages Europe 2 and Africa 3, which are known to circulate in birds in Germany. This case report highlights the importance of USUV as a potential diagnosis for neurological impairments in marine mammals and documents the first cases of USUV infection in harbor seals.

## 1. Introduction

Usutu virus (USUV, *Orthoflavivirus usutuense*) is a zoonotic arbovirus transmitted by mosquitoes. It is closely related to West Nile virus (WNV, *Orthoflavivirus nilense*). USUV was first isolated in South Africa and has been circulating on this continent among wild birds for a long time, apparently without causing high mortality rates [1]. In contrast, massive die-offs of Eurasian Blackbirds (*Turdus merula*) in Austria preceded the initial detection of this virus on the European continent in 2001 [2]. It was discovered later that considerable bird fatalities in Italy during the late 1990s had already been linked causally to the introduction of USUV in 1996 [3]. Subsequently, USUV spread rapidly throughout Europe. It was first discovered in Germany in 2010 in mosquitoes in Baden-Wurttemberg [4] and circulated thereafter for several years in various regions in southern and southwestern Germany until it was detected in almost all of Germany in 2018 [5,6,7]. Phylogenetic studies revealed the circulation of various USUV lineages in birds in Germany, with USUV lineages Europe 2 and 3 and Africa 2 and 3 exhibiting pivotal roles [7,8]. As with WNV, the vectors of this virus are mosquitoes, primarily from the *Culex pipiens* complex. Birds act as reservoir and amplification hosts, while mammals serve as incidental hosts and can also become infected [9]. Mass deaths of birds, especially Eurasian Blackbirds, have been reported following infections with USUV [3,10,11].

USUV infections can cause multisystemic diseases in birds, which are associated with non-specific clinical signs such as apathy and/or immobility and neurological deficits such as ataxia, torticollis or tremors [12]. Encephalitis, multiorgan congestion, renal tubular epithelium necrosis and hemosiderosis in the liver and spleen have been described in Song Thrushes (*Turdus philomelos*) in Spain [13]. USUV is mainly associated with significant outbreaks in passerines, but clinical disease and histopathological alterations have also been described in other avian species. Experimentally infected canaries developed tissue lesions in the brain (neuronal damage and inflammation), in the liver (inflammation, necrosis) and the spleen (inflammation, necrosis, lymphoid tissue degradation), as well as in the lungs (mild inflammation), in the proventriculus (severe inflammation and bleeding), and in the lacrimal glands (necrosis and inflammation) [14]. It should be noted that (histo-)pathological lesions of USUV-associated disease have only been described in birds so far, although infections have been detected in various animals, such as horses, dogs, wild boars, wild ruminants, squirrels, lizards, bats and even humans. In those, clinical signs are usually limited to fevers or rashes, but neurological deficits are also possible [12]. In humans, neuroinvasive cases of USUV infection have been detected in Italy, Austria, Croatia, the Czech Republic, France and Hungary. USUV is also regularly found in asymptomatic blood donors [15,16]. Furthermore, USUV antibodies have been found in a variety of horses without clinical signs, in, e.g., Germany [17,18] and Croatia [16,19].

Severe outbreaks of non-suppurative encephalitis in marine mammals have also been described for other viral infections, e.g., distemper virus [20] or highly pathogenic avian influenza virus [21]. The susceptibility of these animals to arthropod-borne viral infections was recently demonstrated by the detection of a Batai virus infection in a harbor seal (*Phoca vitulina*) in northern Germany [22]. Moreover, there have been some reports of WNV infection in marine mammals worldwide, e.g., when a harbor seal in New Jersey was found with polioencephalomyelitis caused by WNV in 2006 [23]. Another case report described WNV in a killer whale (*Orcinus orca*) in Texas in 2007, where the animal died without any apparent clinical signs but showed WNV-associated pathological lesions [24]. In Germany, WNV has been found in captive harbor seals in Berlin in 2022 and more recently in a zoological facility in Schleswig-Holstein in 2024 [25,26,27]. However, there have been no reports of USUV infections in marine mammals to date.

This case report describes the lethal progression of USUV infections in sick harbor seal pups and the subsequent monitoring of 37 healthy harbor seal pups from the same rescue center located on the North Sea coast in Germany shortly before their release into the wild, two of which were found to be USUV-positive. These are the first case reports of fatal USUV infections in mammals, including descriptions of the clinical course of the disease and the associated (histo-)pathological lesions.

## 2. Case Presentation

Here, we report a USUV outbreak in harbor seal pups at a rescue center at the North Sea coast in the most northern federal state of Germany, Schleswig-Holstein. Five pups died or had to be euthanized due to animal welfare reasons in July and August 2024 after developing severe generalized disease within a remarkably short timeframe including neurological signs. Subsequently, all five harbor seals were necropsied and analyzed using histopathology, revealing signs of (meningo-)encephalitis. USUV RNA was found in the brain and pools of the liver and heart of three of five harbor seals using USUV reverse transcription quantitative real-time polymerase chain reaction (RT-qPCR). Virus-specific immunohistochemical staining (IHC) was performed on these three USUV-RNA-positive animals, detecting USUV antigen in the brain stem and cerebrum. Additionally, blood samples of 37 clinically healthy harbor seal pups raised at the rescue center were tested at a later date in the same year, shortly before their release into the wild. Two of these 37 animals tested positive for USUV RNA as well as USUV-specific antibodies.

### 2.1. Case History and Clinical Findings

In July (*n* = 2) and August (*n* = 3) of the year 2024, neurological signs were observed in five harbor seals at the Seal center Friedrichskoog, a rescue center in Schleswig-Holstein. These animals were assigned case numbers 1 to 5 in chronological order of diagnosis. Case 1 had been staying in the rescue center for two and a half weeks when clinical signs appeared, while the cases 2 to 5 had been fostered at the rescue center for at least one month before clinical sign onset. All of these harbor seals (5/5) showed severe neurological signs, including lethargy, muscle tremor, uncoordinated movement, facial or eye twitching, fin flapping, and loss of body tension. Abnormal behavior such as floating on the water surface, reduced responsiveness, and water avoidance were observed in several cases, suggesting progressive central nervous system (CNS) impairment. Meanwhile, non-neurological clinical signs were infrequent. Elevated body temperature, for example, was documented in two animals (cases 1 and 3). Moreover, forced breathing was documented in two cases (cases 3 and 4). All individuals either died (3/5) or had to be euthanized (2/5) within 30 h following the onset of neurological signs, indicating an acute disease course. Table 1 summarizes relevant information regarding the clinical signs.

### 2.2. Gross Examination

Necropsy was perfomed less than 24 h post mortem at the Landeslabor Schleswig-Holstein in Neumünster. The harbor seals were in moderate (case 1) to good (cases 2 to 5) body condition. There were no consistent macroscopic findings despite depletion of lymphoreticular tissues such as thymus and different lymph nodes. The results can be found in Table 2.

### 2.3. Histopathological Findings

Samples from the lungs, pulmonary lymph nodes, thymus, heart, liver, spleen, kidney, esophagus, stomach, intestine, mesenteric lymph nodes, urinary bladder, muscle, and bone marrow were fixed in 10% buffered formalin and routinely stained with haematoxylin–eosin (HE).

**Figure 1:**
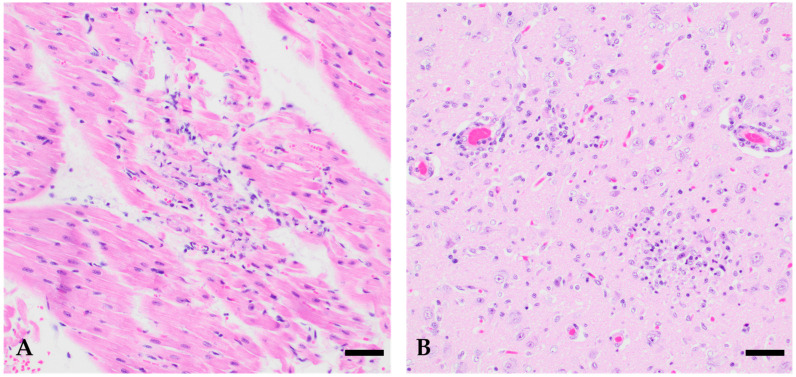
Histomorphological alterations caused by USUV in the heart and brain of two seals, (**A**) heart of case 4, with focal mild, acute, necrotizing, non-suppurative myocarditis, HE, (**B**) cerebrum of case 4 with signs of acute, non-suppurative, necrotizing encephalitis composed of multifocal mild perivascular lymphohistiocytic cuffing and glial nodules with central necrosis, HE, bar 20 µm.

In histopathology, the harbor seals showed a variable pattern of inflammation and degeneration of the brain (Table 2). Case 1 (1/5) showed subacute, suppurative meningoencephalitis, which was most prominent in the meninges of the cerebrum and cerebellum and included the adjacent gray matter but without severe involvement of the white matter. On the other hand, cases 2 to 5 (4/5) showed acute non-suppurative encephalitis of gray and white matter with diencephalon, including basal ganglia and thalamus, as well as the cerebral cortex as the most frequently affected regions. Multifocal perivascular cuffing caused by mononuclear inflammatory cells was present in these four animals (4/4). In addition, predominantly multifocal clusters of glial cells (4/4), neuronal necrosis (4/4) from single neuronal cells (1/4) up to foci of malacia (3/4) were frequent findings. Moreover, signs of non-suppurative vasculitis were seen in two of these harbor seals (2/4). Additional findings most probably related to a viral infection were confined to the heart, showing in three harbor seals (cases 3–5) multifocal mild single-cell necrosis (2/3) up to acute mild non-suppurative necrotizing myocarditis (1/3). Images of the HE-stained heart and brain of case number 4 can be found in Figure 1.

### 2.4. Molecular Differentiation and Phylogeny

RT-qPCR was performed on tissue samples from all five clinically diseased harbor seals to reveal potential viral infections, including USUV and WNV. For the initial detection of specific USUV genome, a protocol was perfomed to detect the non-structural protein 1 (NS1) gene [4]. In a second step, USUV RNA presence was confirmed in positive samples using a protocol targeting the viral non-structural protein 5 (NS5) gene of USUV [28]. The results revealed the presence of USUV RNA in the brain and pools of the liver and heart of 3/5 harbor seals (Table 3). The respective cycle threshold (Ct) values ranged from 16.65 to 37.09 for USUV RT-qPCRs. WNV infections were excluded in all five animals according to an RT-qPCR protocol using WNV-specific 5′ non-translated region primers and probe [29]. The animals with case number 1 and 2 (2/5) tested negative for both USUV and WNV according to RT-qPCR. Therefore, USUV was likely the causative agent in three of the five affected harbor seals showing similar clinical signs, while WNV was not implicated in the disease.

Additional PCR assays and bacterial cultures were performed to rule out other potential pathogens as relevant differential diagnoses: All harbor seals (5/5) tested negative for avian influenza virus, canine herpesvirus and phocine herpesvirus 1, canine and phocine distemper virus, and *Brucella* spp. using PCR. Bacterial cultures of meningeal swabs were sterile (2/5) or only ubiquitous bacteria deemed non-pathogenic were isolated (3/5). Additionally, two harbor seals (cases 1 and 2) were screened for rabies and parvovirus infection by PCR with negative results.

Two organ samples from cases number 3 (brain) and 5 (organ pool of liver and heart) were chosen for USUV lineage classification via viral whole-genome sequencing (WGS) due to their comparatively low Ct values. The extracted RNA was reverse transcribed with non-specific primers and afterwards, only the USUV-derived complementary DNA was amplified using two separate sets of oligonucleotide primers [30] to generate overlapping amplicons in two separate reactions. Amplicons were subsequently processed for WGS using MinION technology (Oxford Nanopore Technologies, Oxford Science Park, Oxford, UK) as previously described by Schwarzer et al. [31]. Consensus sequences were generated using the VirDetector pipeline [32]. Phylogenetic analysis was conducted in MEGA X version 10.2.6 [33] using a maximum likelihood method with 1000 bootstrap replicates and KC754958 as the outgroup. Trees were visualized in FigTree v1.4.4 [34].

**Figure 2:**
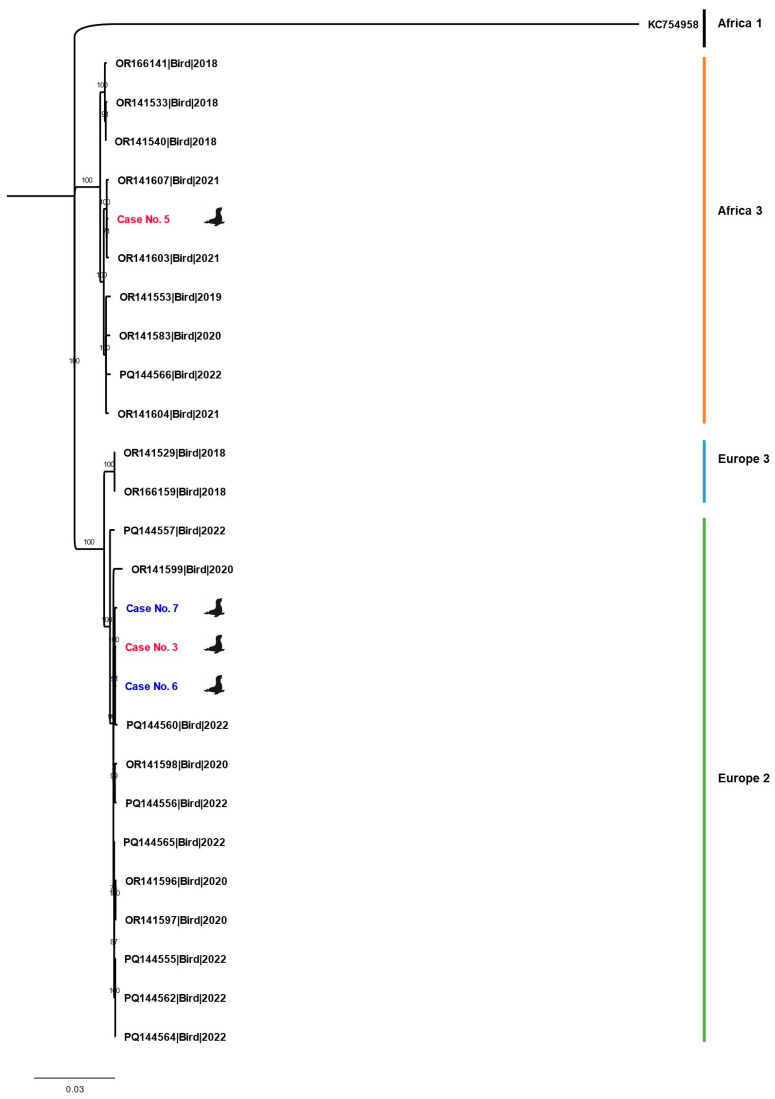
Phylogenetic analysis of USUV lineages from Germany. USUV sequences of harbor seals with clinical signs are shown in red, USUV sequences of harbor seals from the monitoring panel without clinical signs are shown in blue. A maximum likelihood tree was generated using complete genome sequences, with a sequence from the Central African Republic designated as the outgroup. The scale bar represents the mean number of nucleotide substitutions per site. Branch labels indicate bootstrap support values. Sequences obtained in this study are highlighted in red and blue. Detailed information for all sequences is provided in Supplementary File (Table S1).

The analysis for Figure 2 included sequences from USUV-infected birds in Germany from the years 2018 to 2022 [7,35,36]. The phylogenetic analysis was primarily intended for lineage assignment and contextual classification within known circulating German USUV strains. It was not designed to reconstruct transmission dynamics or perform time-resolved phylodynamic analyses, particularly in view of the currently limited number of available mammalian-derived USUV whole-genome sequences. Sequences were selected based on their proximity to the rescue center and supplemented with sequences from the USUV lineage Europe 2 to classify the harbor seal sequences from this study that belong to this lineage. Sequences gained in this study are available under https://doi.org/10.5281/zenodo.18402969. Lineage analyses revealed the harbor seal with case number 5 being infected with a USUV lineage Africa 3 strain while the whole-genome sequence of the harbor seal with case number 3 was found to belong to USUV lineage Europe 2.

### 2.5. Immunohistochemistry

For the three clinically apparent USUV-RNA-positive harbor seals (cases 3 to 5), paraffin blocks were cut (3 µm sections) for immunohistochemical staining with an in-house polyclonal rabbit anti-USUV antibody (pab U433, two h at room temperature). To exclude a WNV infection, consecutive sections were stained with an in-house polyclonal anti-WNV antibody (pab OM8, two h at room temperature). Negative control sections were incubated with goat serum alone. The pretreatment for both antibodies included a blocking step for the endogenous peroxidase using 3% H_2_O_2_/methanol (30 min) and for unspecific reactions using goat serum (1:1 diluted with Tris-buffered saline, 10 min). The USUV antigen was retrieved using a heat pretreatment in the microwave (600 W for 10 min), whereas the WNV antigen was retrieved using PK digestion (37°C, 15 min, 10 mg/ml). The EnVisionTM reagent (Dako) was used as a secondary antibody for 30 min. The slices were finally developed in diaminobenzidine tetrahydrochloride (Fluka) and counterstained with Mayer’s haematoxylin.

**Figure 3:**
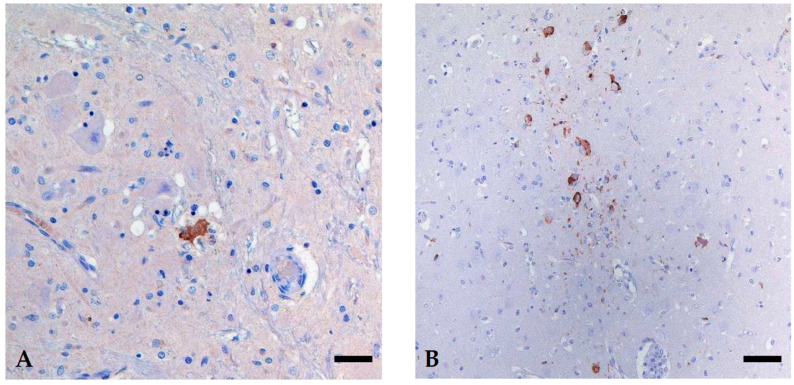
Immunohistochemical detection of USUV antigen in the brain of two seals, (**A**) brain stem of case 3, showing a distinct staining reaction in a necrotic neuron surrounded by glial cells (satellitosis), (**B**) focal cluster of USUV-positive neurons and glial cells in the gray matter of the cerebrum of case 5. In-house polyclonal rabbit anti-USUV antibody (pab U433), bar 20 µm.

In each of these three animals, the cerebrum, brain stem, and cerebellum were investigated. Only two of the investigated animals (2/3) showed a staining reaction for USUV antigen in only a few cells. In case 3, the USUV antigen was detected in single neurons of the brain stem, accompanied by mild glial reactions (satellitosis) and/or signs of necrosis in the affected areas (Figure 3A). In case 5, a distinct reaction pattern with a multifocal cluster of IHC-positive neurons and glial cells was seen in the cerebrum (Figure 3B), while only a single neuron stained positive in the brain stem. No viral antigen was detected in the cerebellum. For case 4, no virus antigen was discernible. In all cases, no cross-reactive binding was detected by using the WNV-specific antibody.

### 2.6. Further Monitoring of Young Harbor Seals in the Rescue Center

In addition to the five animals presented in the preceding paragraphs, of which three were USUV-RNA-positive, blood samples from 37 harbor seal pups without clinical signs raised in the rescue center were taken and tested for USUV antibodies and genomes prior to the release of these animals into the wild in autumn. The tests were conducted two to three months after the discovery of the five harbor seals with the neurological signs described above. Two orthoflavivirus antibody-specific Enzyme-Linked Immunosorbent Assays (ELISAs; ID Screen^®^ Flavivirus Competition, IDvet and INgezim WEST NILE Compac, Ingenasa) were performed according to the manufacturer’s instructions. Two sera (2/37, assigned case numbers 6 and 7) tested positive in both ELISAs, while the remaining 35 sera tested negative. These two positive sera were differentiated by virus neutralization tests (VNTs) for WNV, USUV, and tick-borne encephalitis virus (TBEV). VNTs were conducted as described by Seidowski et al. and Ganzenberg et al. [17,37]. The VNT results revealed neutralizing USUV-specific antibodies (ND_50_ 1:320 and 1:480, see Table 4), but no antibodies specific to TBEV or WNV in the sera of both animals. Furthermore, RT-qPCRs targeting the NS1 [4] and NS5 regions of USUV [28] and the 5′ non-translated region of WNV [29] were performed on the blood of all 37 young harbor seals. RT-qPCRs were also positive for the two serologically USUV-antibody-positive harbor seals and the WNV RT-qPCR was negative. Blood cruor of these two harbor seals was submitted to WGS for USUV lineage classification as described in Section 2.1 of this case report. USUV lineage analysis revealed that the whole-genome sequences of these harbor seal pups belonged to USUV lineage Europe 2, as was also the case for the clinically affected harbor seal number 3 (Figure 2).

## 3. Discussion

Since arboviruses have been circulating among birds in Germany for several years, regular transmission to the mammalian population has been documented each year through reported cases of disease in horses and humans [38,39]. Long-term monitoring networks have been established to track the spread and evolution of zoonotic arboviruses in birds in Germany with a focus on USUV and WNV [6,36]. Case reports remain a crucial component as well for understanding infection chains and virus spread. They also provide information for risk assessments for both animal and public health. In Germany, USUV is widespread throughout the country [36]. Favorable climatic conditions, such as warm, humid springs and hot summers, can favor virus replication especially in large mosquito populations due to ideal breeding conditions. This elevated infection pressure can lead to more frequent transmissions to humans, e.g., as seen in Europe in 2018 [40]. Similar conditions in Germany in 2024 also led to a high mosquito abundance, which resulted in a sharp increase in the reproduction and spread of mosquito-borne arboviruses, many of which were USUV cases in birds in the northwest [31,41,42].

The fact that non-avian species such as humans, horses, dogs, wild boars, wild ruminants, rodents, squirrels, and bats have been described in the past as accidental hosts for USUV [43,44,45] suggests that other mammals, such as harbor seals, can also become accidental hosts. In rare cases, WNV infections have already been reported in harbor seals and killer whales [23,24,26]. Other orthoflaviviruses such as Japanese encephalitis virus (JEV) [46,47,48] and St. Louis encephalitis virus (SLEV) [49] have been detected in marine mammals as well. This exemplifies that marine mammals, and harbor seals in particular, are at risk of exposure to orthoflaviviruses and suggests that USUV could also play a similar role due to the close viral relationships and transmission cycles of WNV and USUV.

Here we present USUV infection in five harbor seals. To the best of our knowledge, this is not only the first description of a USUV infection in marine mammals, but also the first description of a fatal USUV infection in mammals. While arbovirus infections like WNV, SLEV and JEV are already known as a cause of neurological disease in marine mammals, our results clearly indicate that USUV should also be included in standard diagnostic protocols of neurological disease in marine mammals. A rescue center provides all the conditions necessary for care staff to promptly notice behavioral changes, neurological abnormalities, and other clinical symptoms in the animals. Hence, the diagnosis of the first cases in a rescue center rather than in the wild is most likely due to the fact that the animals can be observed more closely there than their counterparts in the wild. It is probable that harbor seals exhibiting neurological signs might perish without detection by humans in open waters.

For the scope of comprehensive documentation, it should also be noted here that in the same summer three additional harbor seals were investigated at Landeslabor Schleswig-Holstein, showing similar pathomorphological and histopathological signs as reported in this case report. This includes in particular non-suppurative necrotizing meningoencephalitis and vasculitis, necrotizing myocarditis and/or necrotizing splenitis. However, only one of these animals showed a clear USUV PCR signal, while the second and third cases were inconclusive and USUV PCR-negative. It is therefore tempting to speculate that the number of unreported cases is significantly higher than the five animals presented here, especially since two pups showed a subclinical infection.

Hence, for a complete diagnostic picture of viral infection, the combination of various methods is highly recommended. Such a comprehensive approach was taken in the present study using clinical findings and histopathological methods, assisted by virological and molecular testing, serological assays and phylogenetic analysis on obtained virus genomes. Most interestingly, the monitoring of the young harbor seals also underlines the possibility of an asymptomatic distribution of USUV infections in marine mammals as two of the 37 young healthy-looking harbor seals tested positive for USUV RNA and antibodies.

In birds, both WNV and USUV infections are very similar, causing a multisystemic disease including clinical signs that range from non-specific clinical signs such as the inability to fly up to neurological signs [12,50]. Histomorphological alterations in birds are also variable in distribution and degree of alteration, but frequently include necrotizing inflammations in the CNS, spleen, heart and liver [7,51,52]. In mammals, however, USUV infection has so far only been described in non-fatal human cases: accidental infections are usually mild, but USUV can also show clear neurovirulence inducing fever, skin rash, and, in rare cases, life-threatening meningoencephalitis and peripheral paralysis [15,44]. This is in accordance with the main findings and the most likely cause of death or severe clinical signs leading to the euthanasia of the harbor seals, which were mostly confined to the CNS. The alterations of the heart, on the other hand, are only mild. These results indicate a strong neurotropism of USUV similar to avian infections. Moreover, case reports of WNV in marine mammals include subacute vasculitis and non-suppurative encephalitis in a killer whale [24] and non-suppurative necrotizing encephalomyelitis in harbor seals [23,26]. However, we must bear in mind that only a few fatal case reports in (marine) mammals exist, hence a final conclusion for host-specific disease manifestation in marine mammals cannot be drawn. Nevertheless, these data not only suggest that USUV and WNV have a similar pathogenesis resulting in analogous diseases [53], but more importantly underline the significance to always include both viruses in differential diagnosis, not only in birds.

One question that remains unanswered is how the harbor seals could have become infected with USUV. The transmission route of orthoflaviviruses in marine animals has not yet been elucidated [54]. In the cases presented here, mosquito bites are the most likely route of infection. At first glance, the skin texture of harbor seals, with a thick epidermis and subcutaneous fat layer, seems almost impenetrable for a mosquito bite, but the animals have thinner skin on their eyelids, nostrils, fin edges, and around the mouth area [55]. This theory is further supported by the fact that these were young harbor seals, which have thinner skin than adult animals. A mosquito could bite while the seal is resting on land during the warm summer months (e.g., in August, as with the animals in the present study). In addition, mosquito exposure in Germany was high in 2024 due to favorable weather conditions [42]. Moreover, vectors are often found in resting places frequented by harbor seals. Incidentally, the staff at the rescue center noted a very high mosquito incidence throughout the entire area over many years. Another hypothetical infection route would be by oral ingestion. Oral transmission for WNV through the uptake of infected small passerines was assumed in rare cases in birds of prey. However, this route is considered to be less effective compared to mosquito bites [50,56,57]. Likewise, experimental infection in birds has been demonstrated intranasally and horizontally via sputum [58], and blackbirds also shed USUV after experimental infection [59]. While fish, the primary food source for captive harbor seals, are not known to be a reservoir for USUV or WNV, oral transmission via the ingestion of dead birds or bird droppings cannot be ruled out as an infection route. Interestingly, vector-independent transmission through contact with feces-contaminated water was already suspected in the transmission of WNV between crocodile hosts [60]. However, orthoflaviviruses are not stable in sea water [54], making this transmission route not very likely.

Phylogenetic analyses revealed that multiple USUV lineages can circulate in the same location. Three of the four whole-genome-sequenced samples belong to USUV lineage Europe 2, which has been circulating in Germany since 2018 and has so far only been found in birds in eastern, central, and southern Germany [35,36]. However, it has not previously been detected near the North Sea coast of Germany, where the harbor seal cases are located. The further spread of lineage Europe 2 in Germany, which was demonstrated here, had already been suspected in previous years [36]. The whole-genome sequence of case number 5, which exhibited clinical signs at the same time as case number 3, belongs to a different USUV lineage: Africa 3. This lineage has been circulating in Germany since 2014 [35] and has already been detected in birds near the rescue center several years before [7,35,36]. This finding shows the parallel and independent co-circulation of USUV lineages and underscores the importance of WGS in elucidating their genetic diversity.

## 4. Conclusions

This report offers the first description of USUV infections and their associated pathology in marine mammals worldwide by combining molecular, serological, histopathological, and phylogenetic methods. The most prevalent clinical signs in the affected animals were neurological signs such as muscle tremor, fin flapping, uncontrolled movements and histopathological alterations, including non-suppurative, in some cases necrotizing, (meningo-)encephalitis partly accompanied by non-suppurative vasculitis as well as necrotizing myocarditis as main lesions. However, USUV infection can also be asymptomatic in this species, as demonstrated by two clinically inapparent harbor seal pups found to be USUV-positive. Four USUV-RNA-positive samples were subjected to whole-genome sequence analysis. Sequences obtained from two of the necropsied animals belonged to USUV lineage Europe 2 and Africa 3, while both of the clinically unsuspicious harbor seals from the monitoring panel belonged to USUV lineage Europe 2. WGS is a useful tool to identify co-circulating USUV lineages and yields good results for expanding knowledge about USUV infections in accidental hosts and lineage distribution. Taken together, it should be kept in mind that arboviruses including USUV can also infect marine mammals, especially during the peak mosquito season, potentially leading to neurological impairments and a fatal outcome and has to be included in differential diagnosis in further examinations.

## Figures and Tables

**Table 1:** Clinical findings in the five harbor seals.

Case No.	Estimated Age	Sex	Neurological Signs	Other Major Clinical Findings	Additional Remarks
1	4 weeks	f	Alternating lethargy and activity, lack of body tension, eye twitching, disorientation	Elevated body temperature	Found dead after 30 h
2	6 weeks	m	Muscle tremor, lethargy, cramping, staggering, fin flapping		Euthanized 30 h after onset of clinical signs
3	7.5 weeks	f	Floating in the pool, lack of body tension, eyes rolled back, fin flapping, head movements	Elevated body temperature, forced breathing	Found dead the next day after onset of clinical signs
4	7.5 weeks	m	Uncoordinated movements, facial twitching, limp lying, cramps	Forced breathing	Found dead the next day after onset of clinical signs
5	7 weeks	f	Eye twitching, head movements	Avoiding water, no position change	Euthanized the same day

m = male; f = female.

**Table 2:** Summary of the results obtained by gross examination, histopathology and immunohistochemistry.

Case No.	Gross Examination				Histopathology		USUV IHC
	LRS	RS	GIT	CNS	CNS	Heart	
1	Severe thymic atrophy; moderate hypoplasia of mesenteric LN	No specific findings	Stomach filled; catarrhal enteritis	No specific findings	Cerebral, cerebellar cortex: multifocal, moderate, subacute suppurative meningoencephalitis	No specific findings	Not done
2	Severe thymic atrophy	Congestion of tracheal mucosa	Stomach slightly filled	Diffuse leptomeningeal edema	Brain stem, diencephalon: multifocal, severe, acute non-suppurative, perivascular to coalescing encephalitis with severe, focal gliosis and neuronal necrosis	No specific findings	Not done
3	Moderate thymic atrophy	Lungs: slightly engorged (less collapsed)	Stomach slightly filled	No specific findings	Diencephalon, basal ganglia, cerebral cortex: multifocal, moderate, acute non-suppurative, perivascular encephalitis with severe, focal gliosis and extensive neuronal necrosis (malacia)	Multifocal single-cell necrosis	Brain stem: single pos. neurons
4	Severe thymic atrophy	Congestion of tracheal mucosa	Stomach filled	Injected meningeal blood vessels; red, greasy material in the basal meninges	Hippocampus, thalamus, cerebral cortex: multifocal, moderate, acute non-suppurative, perivascular encephalitis and vasculitis with moderate, focal gliosis and extensive neuronal necrosis	Multifocal, mild, acute myocardial necrosis	Negative
5	Severe thymic atrophy; severe hypoplasia of axillary, inguinal, mesenteric LN	Congestion of tracheal mucosa; lungs: severe acute congestion and edema	Empty stomach	Diffuse leptomeningeal edema	Cerebral cortex: multifocal, severe, acute non-suppurative, perivascular meningoencephalitis and vasculitis with bleeding diathesis, severe, focal gliosis and extensive neuronal necrosis accompanied by a mild infiltration of neutrophilic granulocytes	Multifocal, acute, myocardial single-cell necrosis	Cerebrum: multifocal cluster of pos. neurons/glial cells; brain stem: single pos. neurons

LRS = lymphoreticular system; LN = lymph nodes; RS = respiratory system; GIT = gastrointestinal tract; CNS = central nervous system; IHC = immunohistochemistry; pos. = positive.

**Table 3:** Molecular test results of the five dead harbor seals.

Case No.	Tested Tissue	Ct Value USUV NS1 RT-qPCR	Ct Value USUV NS5 RT-qPCR	Ct Value WNV 5′NTR RT-qPCR
1	B/LH	>42/>42	>42/>42	>42/>42
2	B/LH	>42/>42	>42/>42	>42/>42
3	B/LH	16.65/33.08	17.63/33.32	>42/>42
4	B/LH	>42/>42	41.19/32.76	>42/>42
5	B/LH	37.09/>42	34.72/30.83	>42/>42

B = brain; LH = organ pool of liver and heart; Ct = cycle threshold; Ct value >42 = negative.

**Table 4:** Molecular and serological test results from two clinically healthy USUV-positive harbor seal pups from the rescue center.

	Virology			Serology				
	RT-qPCR Blood			ELISA		VNT		
Case No.	Ct Value USUV NS1 RT-qPCR	Ct Value USUV NS5 RT-qPCR	Ct Value WNV 5′NTR RT-qPCR	ID SCREEN^®^ Flavivirus Competition [S/N%]	INgezim WEST NILE Compac [S/P%]	USUV ND_50_	WNV ND_50_	TBEV ND_50_
6	40.04	35.99	>42	pos. [19.58]	pos. [84.09]	1:320	1:20 *	neg. (<1:10)
7	33.89	35.69	>42	pos. [12.28]	pos. [84.55]	1:480	neg. (<1:10)	neg. (<1:10)

ND_50_ = 50% neutralization dose; pos. = positive; neg. = negative; * = cross-reactive; Ct value >42 = negative.

## Data Availability

The original contributions presented in this study are included in the article and its Supplementary Material. Sequences gained in this study are openly available under https://doi.org/10.5281/zenodo.18402969. Further inquiries can be directed to the corresponding author.

## Supplementary Materials

The following supporting information can be downloaded at: https://www.mdpi.com/article/10.3390/v18030371/s1. Table S1: Information for all sequences of birds from Germany used for phylogenetic analysis (Figure 1).

## Abbreviations

The following abbreviations are used in this manuscript:

CNS	Central nervous system
Ct	Cycle threshold
e.g.	For example (*exempli gratia*)
ELISA	Enzyme-Linked Immunosorbent Assay
HE	Haematoxylin–eosin
IHC	Immunohistochemistry
JEV	Japanese encephalitis virus
ND_50_	50% neutralization dose
No.	Number
NS1	Non-structural protein 1
NS5	Non-structural protein 5
RT-qPCR	Reverse transcription quantitative real-time polymerase chain reaction
SLEV	St. Louis encephalitis virus
TBEV	Tick-borne encephalitis virus
USUV	Usutu virus
WGS	Whole-genome sequencing
WNV	West Nile virus
VNT	Virus neutralization test
